# Application of a Customised Franz-Type Cell Coupled with HPTLC to Monitor the Timed Release of Bioactive Components in Complex Honey Matrices

**DOI:** 10.3390/mps6040070

**Published:** 2023-08-03

**Authors:** Md Lokman Hossain, Minh Nguyen, Leah Benington, Lee Yong Lim, Katherine Hammer, Dhanushka Hettiarachchi, Cornelia Locher

**Affiliations:** 1 Division of Pharmacy, School of Allied Health, University of Western Australia, Crawley 6009, Australia; mdlokman.hossain@research.uwa.edu.au (M.L.H.); minh.nguyen@uwa.edu.au (M.N.); leah.benington@uwa.edu.au (L.B.); lee.lim@uwa.edu.au (L.Y.L.); dhanushka.hettiarachchi@outlook.com (D.H.); 2 School of Biomedical Sciences, University of Western Australia, Crawley 6009, Australia; katherine.hammer@uwa.edu.au; 3 Cooperative Research Centre for Honey Bee Products Limited, 128 Yanchep Beach Road, Perth 6035, Australia

**Keywords:** in vitro dissolution, honey, topical honey-loaded products, dialysis membrane, Franz cell, custom-designed Franz-type diffusion cell, HPTLC, release profile

## Abstract

The aim of this study was to assess the release profile of components in five different honeys (a New Zealand Manuka and two Western Australian honeys, a Jarrah honey and a Coastal Peppermint honey) and their corresponding honey-loaded gel formulations using a custom-designed Franz-type diffusion cell in combination with High-Performance Thin-Layer Chromatography (HPTLC). To validate the suitability of the customised setup, release data using this new approach were compared with data obtained using a commercial Franz cell apparatus, which is an established analytical tool to monitor the release of active ingredients from topical semisolid products. The release profiles of active compounds from pure honey and honey-loaded formulations were found to be comparable in both types of Franz cells. For example, when released either from pure honey or its corresponding pre-gel formulation, the percentage release of two Jarrah honey constituents, represented by distinct bands at R_F_ 0.21 and 0.53 and as analysed by HPTLC, was not significantly different (*p* = 0.9986) at 12 h with over 99% of these honey constituents being released in both apparatus. Compared to the commercial Franz diffusion cell, the customised Franz cell offers several advantages, including easy and convenient sample application, the requirement of only small sample quantities, a large diffusion surface area, an ability to analyse 20 samples in a single experiment, and lower cost compared to purchasing a commercial Franz cell. Thus, the newly developed approach coupled with HPTLC is conducive to monitor the release profile of minor honey constituents from pure honeys and honey-loaded semisolid formulations and might also be applicable to other complex natural-product-based products.

## 1. Introduction

Drug dissolution testing is essential in medicinal product development and quality control as it is a predictor of in vivo drug behaviour and, thus, ultimately therapeutic results. Dissolution testing was first applied for immediate release solid oral dosage forms and later broadened to controlled/modified release solid oral pharmaceuticals [1]. However, over the span of years the usage of dissolution testing has extended to a range of dosage forms for instance, suspensions, orally disintegrating tablets, chewable tablets, chewing gums, transdermal patches, semisolid topical preparations, suppositories, implants, and injectable microparticulate formulations and liposomes [1,2]. As these formulations have become more prevalent, revised testing methods have also emerged [1,2]. For immediate release oral solid drug products, drug release studies are commonly referred to as ‘dissolution’ tests, as the intent of these formulations is to lead to rapid drug dissolution in the target medium. In the case of topical dosage forms, the test is preferably called a ‘drug release’ or ‘in vitro release’ test [3]. The overall concepts of drug dissolution tests for solid oral dosage forms should also be applicable to several in vitro dissolution/release tests for ‘novel’ or ‘special’ dosage forms [3,4,5,6]. However, as the above mentioned ‘novel’ or ‘special’ dosage forms reveal important variations in formulation design, which ultimately result in diverse physicochemical and release characteristics, it is challenging to develop a single test system which can be applied to study the drug release profile of all special dosage forms [3,4]. Various apparatus, processes, and methods are applied on a case-by-case basis, and the adopted method may be explicit to the dosage form category, the formulation type, or even to a particular individual product [4,5]. Table 1 shows the official dissolution methods for different dosage forms. The United States Pharmacopeia (USP) includes several apparatuses for transdermal systems (TDSs) such as Apparatus 5 (paddle over disc), Apparatus 6 (cylinder), and Apparatus 7 (reciprocating holder). However, there is no stated method for determining the dissolution/release of natural-product-based formulations like honey. The capture of the in vitro release profile of multiple compounds from dosage forms incorporating complex natural products, like honey, is particularly challenging as a suitable, convenient analytical method is required to account for often small concentrations of different compounds in the release medium.

**Figure 1:**
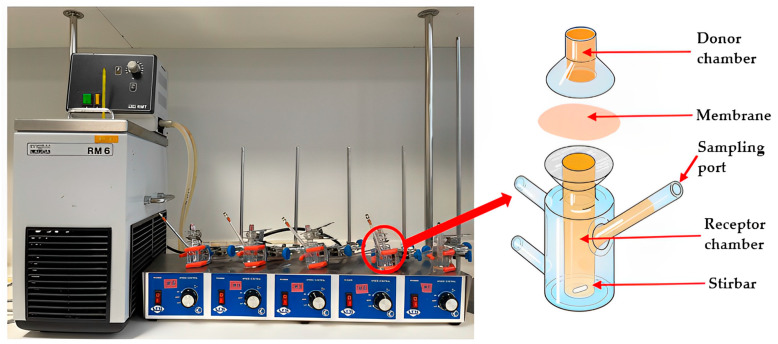
Commercial Franz diffusion cell.

As listed in Table 1, the Franz diffusion cell, which is the official testing apparatus for semisolid dosage forms, might offer a suitable setup for the release study of active constituents from natural-product-based topical semisolid formulations. However, there are some limitations, most notably the relatively large volume of the receptor chamber (Figure 1), which necessitates relatively large quantities of samples to be tested in order to detect various bioactive compounds that are released from the sample’s complex phytochemical constituent profile. In the case of honey, for example, non-sugar constituents, which are associated with many of its bioactivities, for example its antioxidant effects [7], only comprise about 3% of the total weight [8,9,10]. Nonetheless, it is the release of these minor constituents that is of interest and, hence, requires adequate monitoring in release studies on honey and formulations incorporating honey as its active pharmaceutical ingredient (API). Furthermore, the diffusion area created by the dialysis membrane used in the Franz cell is relatively small and, therefore, application of an adequate amount of the semisolid products is challenging. In particular, the introduction of air bubbles while spreading a sticky sample like honey onto the membrane needs to be avoided as they might lead to inconsistent and inaccurate results. This is true for both the commercial and also the customised Franz cells. However, because the surface area of the commercial cell is small, any error associated with the presence of air bubbles is magnified compared to the customised cell, where the area of sample application is larger. A larger membrane surface area, as presented in the customised Franz-type cell developed as part of this study, assists with easier sample application.

**Figure 2:**
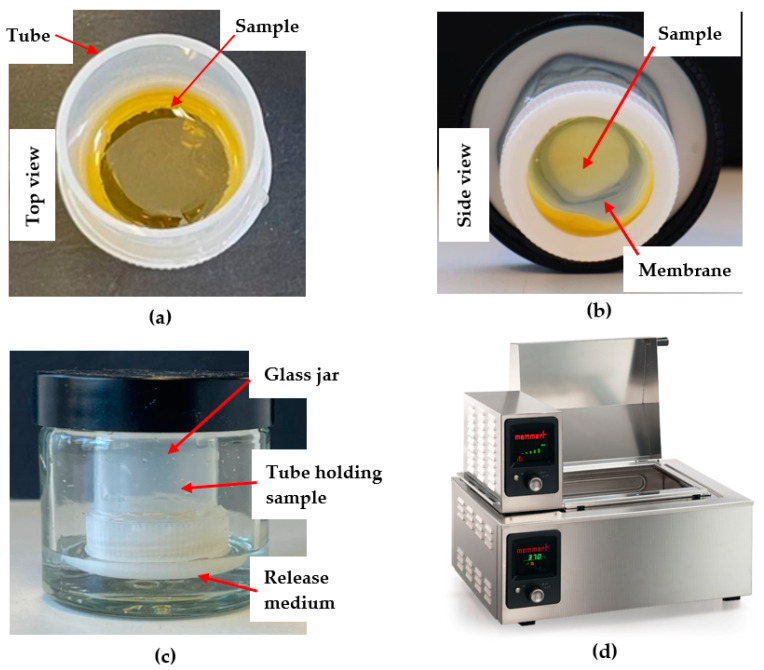
Custom-designed Franz-type diffusion cell: (**a**) Top view of plastic tube (donor compartment); (**b**) Side view of plastic tube (donor compartment); (**c**) Glass jar (receptor compartment) holding donor compartment tube; (**d**) Shaking water bath.

The custom-designed Franz-type diffusion cell (Figure 2) adopts essential components of the Franz cell as it also features a donor and a receptor compartment separated by a dialysis membrane, which mimics a natural barrier such as a mucous membrane. In vivo motion simulated in the Franz cell apparatus by a magnetic stirrer is incorporated in the customised Franz-type cell setup by placing the glass jars holding the sample tubes in a shaking water bath. Like the Franz cell, the customised Franz-type cell setup also allows to mimic body temperature as the water bath temperature is set to 37 °C. An advantage of the customised Franz-type cell setup is the ability to test a large number of samples simultaneously. In this study, fifteen samples were run simultaneously, but the size of the shaking water bath could accommodate up to twenty samples, whereas the Franz cell only allows the running of five samples in parallel. Another difference, already stated earlier, is the ability to use a smaller receptor compartment in the customised Franz-type cell setup, which translates into smaller volumes of receptor fluid and, thus, higher concentrations of APIs for analysis, which is of particular importance when analysing formulations incorporating complex natural products, in this study honey and honey-based formulations, which might contain multiple bioactive compounds present at low concentrations. The customised Franz-type cell setup can also be customised in terms of glass jar size, which will impact on the volume of the receptor compartment as well as the diffusion surface area, and, thus, allows for adaption to specific analysis requirements. The essential features of Franz cell and customised Franz-type cell setup are presented in Table 2.

An additional consideration is the type of instrumentation used to detect and quantify compounds released from complex natural-product-based samples that might contain a multitude of APIs into the receptor compartment. 

High-Performance Thin-Layer Chromatography (HPTLC) is a widely employed semiautomated technique for the chromatographic analysis of pharmaceuticals, natural products, clinical samples, and foodstuffs. It is an enhanced and sophisticated form of thin-layer chromatography consisting of semiautomated sample application, development, visualisation, and data analysis. The usage of HPTLC plates and the capability to regulate and automate critical steps (e.g., sample application, development, and derivatisation) along with entirely automated image analysis allows for qualitative and also quantitative analyses [11,12]. Moreover, HPTLC analyses generate a range of datasets, such as images taken under different light conditions, R_F_ and RGB values of individual bands, and their peak height and peak area. If a TLC Scanner is included in the instrumental setup, UV-Vis and fluorescence spectra of individual bands can also be generated. With this, HPTLC analysis is an ideal approach to monitoring the simultaneous release of multiple constituents, even if their chemical identity is not yet established, as is demonstrated in this study for pure honey and honey-based formulations.

## 2. Materials and Methods

### 2.1. Chemicals and Reagents

4,5,7-Trihydroxyflavanone was obtained from Alfa Aesar, England, UK; Anhydrous sodium sulphate and dichloromethane were purchased from Merck KGaA, Darmstadt, Germany. Spectra/Por^®^ Dialysis Membrane (molecular weight cut-off (MWCO): 3500 Da) was sourced from Repligen, Waltham, MA, USA. Methanol, toluene, ethyl acetate, and formic acid were obtained from Ajax Finechem Pvt Ltd., Sydney, NSW, Australia. NaCl and KCl were sourced from ChemSupply Pty Ltd., Gillman, South Australia and Na_2_HPO_4_ and KH_2_PO_4_ were purchased from Ajax Finechem, New South Wales, Australia. Blu Tack^©^ was obtained from Officeworks, Perth, Australia.

### 2.2. Honey and Honey-Based Formulations

Honey-based formulations were prepared according to a previously published protocol [13]. The honeys used in this study were two Western Australian (WA) Manuka honeys (*Leptospermum* sp.), a WA Coastal Peppermint (*Agonis flexuosa*) and a WA Jarrah honey (*Eucalyptus marginata*), and a New Zealand Manuka honey (*Leptospermum scoparium*). 

The botanical origin, supplier name, and year of the five honeys are shown in Table 3. The identification of the nectar source was based on beekeeper information taking into account the availability of flowering nectar, the honeys’ organoleptic characteristics, and the location of the respective apiaries/hives. In brief, honey loaded gel solutions were prepared by incorporating pure honey into sodium alginate solution. Firstly, using a 100 mL volumetric flask, 2 g (or 3 g in the case of Coastal Peppermint honey preparations) of alginate were dissolved in 60 mL sterile water, followed by the addition of 70 g honey and sterile water to a final volume of 100 mL.

### 2.3. Commercial Franz Cell Diffusion Apparatus

The release study of honey and honey-based products was conducted in a commercial Franz diffusion cell (Scientific Equipment Manufacturers (S.E.M) (SA) Pty. Ltd., Magill, South Australia) following a methodology described by Hossain et al. [14]. The dialysis membrane to be used in the experiment was cut into pieces of 4.5 cm^2^ resulting, once fitted into the apparatus, in a diffusion area of 0.78 cm^2^. The membrane pieces were incubated in phosphate buffered saline (PBS) for 15 min before being mounted between the donor and the receptor chambers of the five Franz cells, all of which were maintained at 37 °C using a temperature-controlled water bath. A total of 5 mL sonicated PBS were added to the receptor chambers and stirred constantly using a magnetic bar (RPM 500). After applying 200 mg of the test samples (pure honeys or the corresponding honey-loaded formulations) on the membrane surfaces, 300 μL samples were withdrawn from the receptor chamber at 15 min, 30 min, 1 h, 3 h, 6 h, and 12 h. After each sample withdrawal, the volume taken from the receptor chamber (300 µL) was replaced with the equal volume of new PBS buffer.

### 2.4. Customised Franz-Type Cell Setup on Shaking Water Bath

The newly developed customised Franz-type cell setup (Figure 2) consisted of a 50 × 27 mm polycarbonate-based transparent tube with screw cap closure (Thermo Fisher Scientific, Waltham, MA, USA) as donor compartment, a glass jar (50 mL) with a plastic screw cap as the receptor compartment and a temperature controlled shaking water bath (Memmert, GmbH+Co.KG, Schwabach, Germany). The dialysis membrane (diffusion area 3.80 cm^2^) was attached to the lower part of the plastic tube using the screw cap in which a circular opening of 22 mm in diameter had been cut (Figure 2b). To allow for a direct comparison of release data obtained from this customised setup with data generated by using the commercial Franz cell, the same sample quantities and solution volumes were used. Thus, 200 mg of the pure honeys and their respective formulations were loaded directly onto the membrane in the donor tube (Figure 2a). The donor compartment was then attached to the cap of the glass jar cap using a putty-like pressure-sensitive adhesive (Blu Tack©). The tube holding the sample was immersed into the glass jar filled with 5 mL of PBS buffer as the release medium (Figure 2c). The container was placed in the shaking water bath (Figure 2d) at a temperature of 37 °C and its shaking motion was set to Level 5 (150 strokes/min) (Figure 2). A total of 300 µL of sample were collected at 15 min, 30 min, 1 h, 3 h, 6 h, and 12 h. After each sample withdrawal (300 µL), the volume taken from the receptor chamber was replaced with the same volume of fresh PBS buffer. 

### 2.5. Preanalysis Sample Preparation

A solution of 4,5,7-trihydroxyflavanone in methanol was prepared (0.5 mg/mL) and used as a reference solution for HPTLC analysis. The respective baseline samples for all honeys and their corresponding formulations (t = 0 min) were prepared as follows: 200 mg pure honey/honey-loaded formulations were dissolved in 1 mL of deionised water followed by three extractions with 5 mL each of a mixture of dichloromethane and acetonitrile (50:50 *v*/*v*) [13]. The extraction efficiency for this approach was determined by spiking artificial honey (made from 1.5 g sucrose, 7.5 g maltose, 40.5 g fructose, and 33.5 g glucose in 17 mL of sterile distilled water) [14] with 0.3 mg of 4,5,7-trihydroxyflavanone and was found to be 99.55%. After the addition of MgCl_2_ anhydrous (approximately 500 mg) to the combined organic extracts and filtration, the solvent was evaporated to dryness using compressed air. The dried extracts were stored at 4 °C. Prior to HPTLC analysis, they were reconstituted in 100 µL methanol. Sample aliquots (300 µL) collected at different time points from the release medium of both release apparatus were extracted in the same way as described above. 

### 2.6. HPTLC Analysis of Released Honey Constituents

A total of 4 µL of the reference solution and 7 µL of each sample were applied onto silica gel 60 F_254_ HPTLC glass plates using the semiautomated sample application device (Linomat 5; CAMAG, Muttenz, Switzerland). The chromatographic separation was performed in an automated development chamber (ADC2, CAMAG) using a mixture of toluene: ethyl acetate: formic acid, 1:6:1 (*v*/*v*) as the mobile phase. The obtained chromatographic results were documented using an HPTLC imaging device (TLC Visualizer, CAMAG) at the wavelengths 254 nm and 366 nm, respectively, followed by automated digital processing and analysis of the obtained chromatographic images using a specialized HPTLC software (visionCATS, CAMAG) [13].

### 2.7. Statistical Analysis

All tests were accomplished in triplicate, and the results were analysed using a one-way analysis of variance (ANOVA) followed by Tukey’s honestly significant difference (TukeyHSD) test. The level of significance was set at 0.05 and a *p*-value of less than 0.05 was reasoned statistically significant. All statistical analyses were accomplished using Microsoft Office 365, GraphPad Prism 9.4.1 (GraphPad Software, San Diego, CA, USA). 

## 3. Results

**Figure 3:**
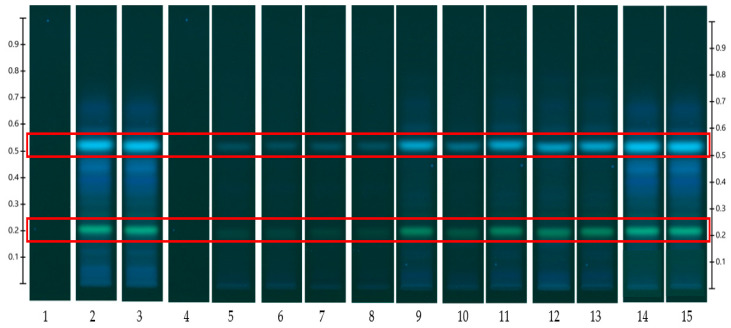
Jarrah (JAR) honey—red box indicates monitored bands at R_F_ 0.21 and 0.53; internal standard: 4,5,7-trihydroxyflavanone (Track 1), system suitability test: JAR honey extract (Track 2), Extract at baseline/0 min (Track 3), extracts obtained from Franz cell and customised Franz-type cell setup at 15 min (Track 4 and 5), 30 min (Track 6 and 7), 1 h (Track 8 and 9), 3 h (Tracks 10 and 11), 6 h (Tracks 12 and 13), and 12 h (Track 14 and 15); image taken at 366 nm.

**Figure 4:**
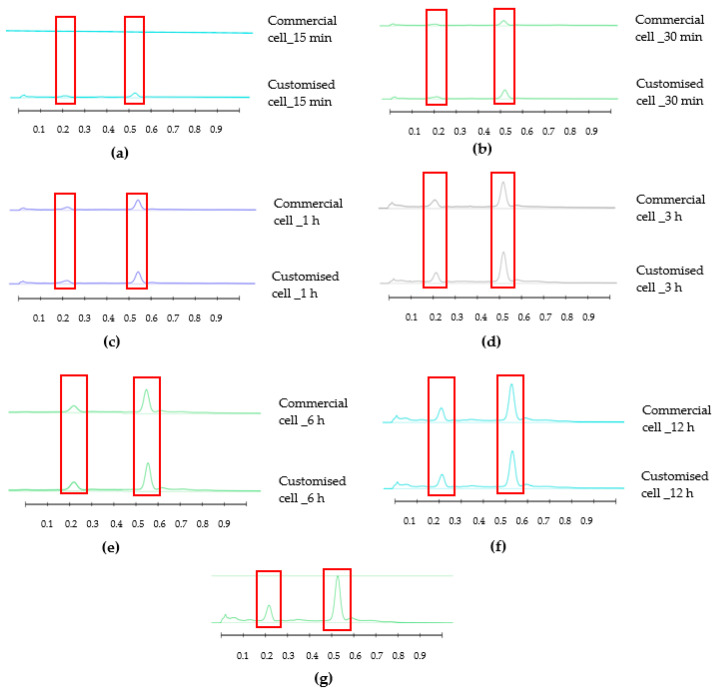
Peak profile of compounds of interest (R_F_ 0.21 and R_F_ 0.53) released from pure Jarrah honey using Franz cell and customised Franz-type cell setup: (**a**) 15 min; (**b**) 30 min; (**c**) 1 h; (**d**) 3 h; (**e**) 6 h; (**f**) 12 h; (**g**) baseline (0 min). Image taken at 366 nm. Red boxes highlight monitored bands.

**Figure 5:**
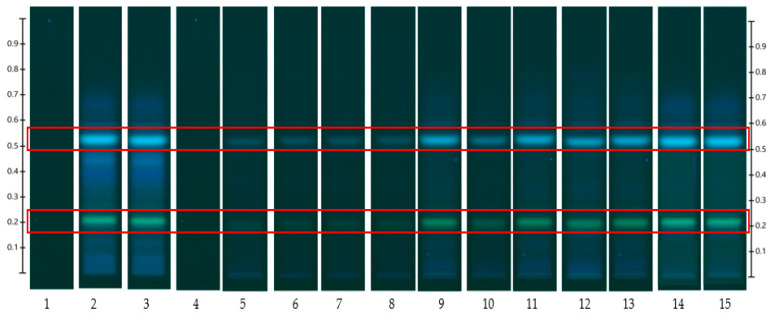
Jarrah (JAR) honey-loaded pre-gel formulation—red box indicates monitored bands at R_F_ 0.21 and 0.53; internal standard: 4,5,7-trihydroxyflavanone (Track1), system suitability test: JAR honey extract (Track 2), extract at baseline/0 min (Track 3), extracts obtained from Franz cell and customised Franz-type cell setup at 15 min (Track 4 and 5), (Track 6 and 7), 1 h (Track 8 and 9), 3 h (Track 10 and 11), 6 h (Track 12 and 13), and 12 h (Track 14 and 15). Image taken at 366 nm.

**Figure 6:**
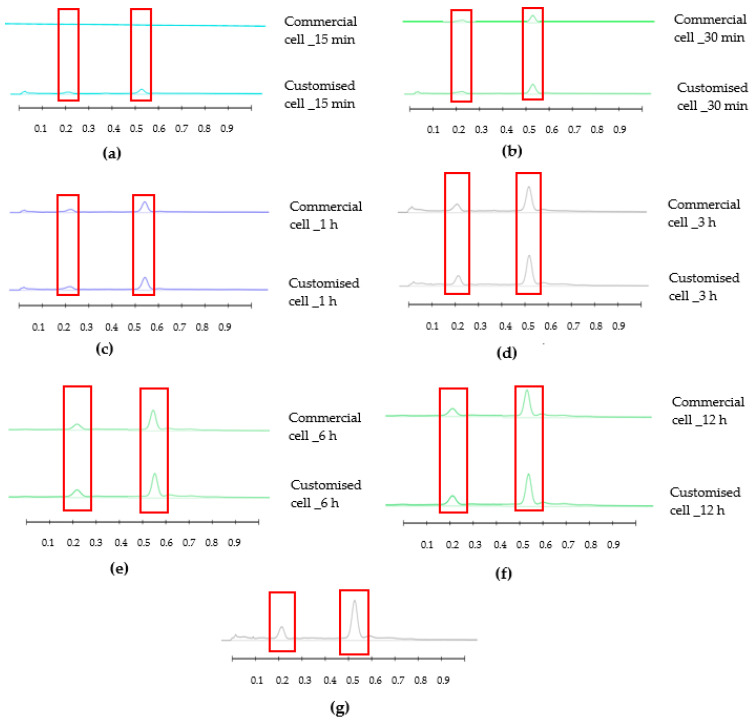
Peak profile of compounds of interest released from Jarrah (JAR) honey pre-gel extract at R_F_ 0.21 and 0.53 using Franz cell and customised Franz-type cell: (**a**) 15 min; (**b**) 30 min; (**c**) 1 h; (**d**) 3 h; (**e**) 6 h; (**f**) 12 h; (**g**) baseline. Image taken at 366 nm. Red boxes highlight monitored bands.

The results obtained from the release study are captured in HPTLC fingerprints (also presented by peak profile) where bands at specific R_F_ and of particular colour represent individual released compounds (Figure 3, Figure 4, Figure 5 and Figure 6). For each honey and its related formulations, at least one distinct compound was monitored over 12 h in both release apparatus and its concentration in the respective receptor compartment, derived from its respective peak area (AU) in the generated peak profile (Figure 4 and Figure 6) was expressed as % release in relation to the corresponding bands and their peak profile areas in the samples prior to the commencement of the release study (baseline/0 min). This allowed for direct comparisons of the % release data for each compound obtained from both release apparatuses.

### 3.1. Pure Honeys

For illustrative purposes, the following section presents the sets of data obtained from the release study of pure Jarrah honey and its pre-gel solution only. 

The HPTLC fingerprints of pure Jarrah honey obtained using the Franz diffusion cell and the customised Franz-type cell setup are shown in Figure 3. Two distinct bands (at R_F_ 0.21 and R_F_ 0.53) were selected for monitoring and chromatograms corresponding to these bands are presented in Figure 4. The release data of the two compounds are shown in Table 3 (% release), Table 4 (% release per unit area of the dialysis membrane), and Table 5 (time to release 25, 50, and 75%). The HPTLC chromatograms of remaining four pure honeys are included in Supplementary File.

### 3.2. Honey-Loaded Pregel Formulations

The HPTLC fingerprints and their corresponding chromatograms of a Jarrah honey-loaded pre-gel sample are shown in Figure 5 and Figure 6, respectively. The HPTLC fingerprints, and chromatograms of the remaining four honey-loaded formulations are presented in Supplementary File. While, again, distinct bands at R_F_ 0.21 and R_F_ 0.53 were monitored in this case, the illustrated approach allows for the capturing of a wide range of potentially bioactive compounds that were released over time from these samples.

**Figure 7:**
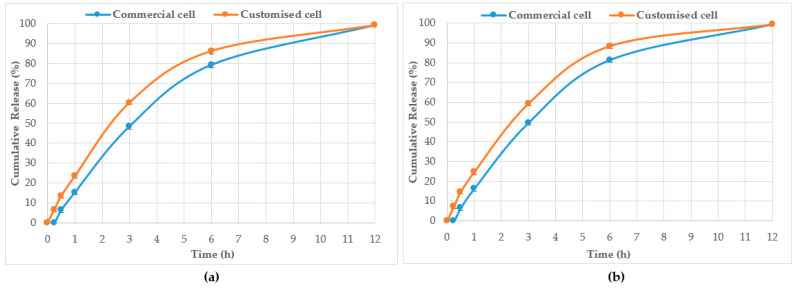
% Cumulative release of compound from pure Jarrah honey at R_F_ 0.21 (**a**) and R_F_ 0.53 (**b**) using the Franz cell and the customised Franz-type cell setup.

**Figure 8:**
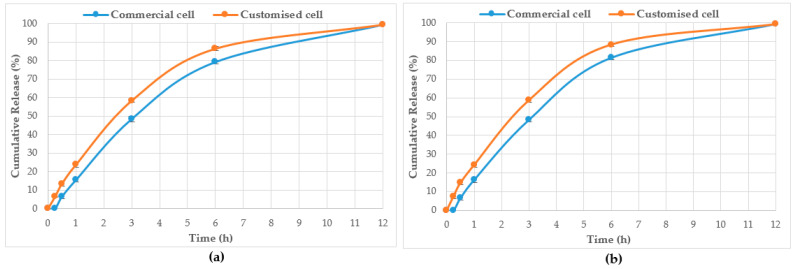
% cumulative release of compound from Jarrah honey pre-gel at R_F_ 0.21 (**a**) and R_F_ 0.53 (**b**) using the commercial Franz cell and the customised Franz-type cell setup.

Table 4 displays the total cumulative % components release whereas Table 5 shows the % components release per unit area of the dialysis membrane. Table 6 shows the dissolution rates (i.e., time taken to release 25%, 50%, and 75% of the baseline values) of select Jarrah honey constituents from pure honey and also its corresponding pre-gel formulation. A comparative % cumulative release of the two compounds (R_F_ 0.21 and 0.53) monitored for their release from pure Jarrah honey in both the Franz cell and the customised Franz-type cell setup is shown in Figure 7. A similar pattern can also be seen for the corresponding Jarrah honey pre-gel formulation (Figure 8). No statistically significant difference (*p* = 0.9986) could be seen in the % cumulative release for pure Jarrah honey and its gel formulation after 12 h.

## 4. Discussion

The in vitro drug release profile is considered an important indicator of in vivo product behaviour and, hence, therapeutic action [15,16]. For all dosage forms, product quality and performance may be measured through numerous in vivo and/or in vitro experiments [17,18]. Because of this, it is greatly desired to measure the release of APIs from pharmaceutical formulations, including semisolid dosage forms [19]. Because of the cost, time, labour, and need for human subjects/animals associated with in vivo pharmacokinetic studies, the collation of in vitro drug release data is a popular surrogate measure for in vivo product performance. The choice of an appropriate release testing method depends on the type of formulation. For example, according to the USP, oral solid dosage forms can be analysed through a dissolution apparatus fitted either with a basket or a paddle stirrer as well as through a reciprocating cylinder or flow-through cell apparatus.

Honey is a super saturated sugary natural substance produced by bees mainly from the nectar of flowers [8,9]. Honey is composed of sugars (approximately 80%), water (about 17%), and ‘other’ constituents (approximately 3%) [8,9,10]. These minor components are believed to be significant in affecting not only the organoleptic characteristics of honeys but also their bioactivity profiles. To date, more than 400 compounds have been reported in honey [13]. When honey is used in its pure form or as a therapeutic agent in a formulation, it is desirable to quantify the release of each of the numerous phytochemicals present in the honey, even though often their chemical identity is not yet known. In this light, the release studies of honey and also honey-loaded formulations are more challenging compared to formulations which contain only a single or a few well defined APIs. 

HPTLC is an ideal analytical approach to monitor the individual constituents from a complex natural mixture like honey as it allows us to visualise the constituents even if they are not yet chemically identified. In this study, using the commercial Franz cell to determine the release of constituents from pure honey and the honey gel solution, it took 30 min for any bands of compounds to be detectable in the sample collected from the receptor compartment (Figure 3, Figure 4, Figure 5 and Figure 6). On the other hand, using the customised Franz-type cell setup developed as part of this study, bands of compounds of interest could be detected as early as 15 min in the case of pure Jarrah honey as well as its gel formulations (Figure 3, Figure 4, Figure 5 and Figure 6). A similar trend can be seen when the % release of a particular compound (R_F_ 0.21) from pure Jarrah honey is considered, with 48.4% detectable at 3 h in the Franz cell system but 60.3% in the customised Franz-type cell setup. The prolongation of incubation time tended to minimize the difference in the release rate, with the % release of that compound reaching more than 99% at 12 h using both methods. This illustrates that the customised Franz-type cell setup allows for a faster release of constituents compared to the Franz cell. This might be due to its larger surface area for release. When the % release was normalised against the surface area available for release (Table 5), it was noticed that, except for the first time point of sample collection (15 min), the percentage release of the monitored honey constituents per unit area of the dialysis membrane was higher at all time points in the commercial Franz cell compared to the customised Franz cell setup. This might be due to the faster stirring rate of magnetic stirring in the commercial cell compared to the motion created in the shaker bath employed for the customised cell, and also, possibly, due to the higher concentration gradient across the release area in the commercial Franz cell given its smaller surface area (0.78 cm^2^ vs. 3.80 cm^2^). Similar trends were seen when comparing the other investigated honeys and their respective pre-gel formulations (see Supplementary Files). These findings suggest that the customised Franz-type cell coupled with HPTLC analysis is effective in monitoring the release pattern of honey constituents and might also be useful for release studies of other formulations incorporating complex natural products as APIs. 

The newly developed setup offers several advantages over the traditional Franz cell system. As mentioned earlier, the Franz diffusion cell allows us to analyse five samples in a single run, whereas the customised Franz-type cell setup permits running 20 samples simultaneously and can be customised to specific analysis requirements. Moreover, it offers a larger surface area for diffusion which makes sample application, assuming the same level of competency when using both apparatuses, much easier and assists in avoiding air bubble formation, which otherwise might interfere with the diffusion leading to inaccurate results [20,21,22,23,24,25]. An additional benefit is related to accessibility because any lab could devise the customised Franz cell whereas not every lab has access to the commercial Franz cell setup. Moreover, while this study was conducted using a complex natural product like honey, it can be assumed that the monitoring of the release of constituents from the modified setup, in particular when coupled with HPTLC analysis, might also be applicable to other natural products incorporating multiple constituents, even at a low concentration. 

## 5. Conclusions

The newly developed customised Franz-type cell setup coupled with HPTLC is capable of monitoring the release profile of honey constituents from the pure honey matrix and also from honey-loaded formulations. It provides several advantages over the commercial Franz diffusion cell system such as a simple and customisable setup, the ability of analysing more samples per run and a large surface area which assists in sample application. This preliminary study suggests that the customised Franz-type cell setup might be useful for analysing a wide range of topical formulations including those which contain complex natural products as has been demonstrated in this study using honey and honey-based semisolid formulations. Thus, the setup might be useful in the research and development of topical products incorporating a complex chemical profile (e.g., multiple APIs at a potentially low concentration) as well as in settings where the routine analysis of the release profile of a large number of semisolid formulations is required.

## Figures and Tables

**Table 1:** Apparatus for drug release testing of various dosage forms.

Dosage Form	Method	USP Apparatus Classification
Oral solid dosage forms	Basket apparatus, paddle apparatus, reciprocating cylinder, or flow-through cell	Dissolution Apparatus 1, Apparatus Type 2, Dissolution Apparatus Type 3, Dissolution Apparatus 4
Oral suspensions	Paddle apparatus	Apparatus Type 2
Oral disintegrating tablets	Paddle apparatus and disintegration method	Apparatus Type 2
Chewable tablets	Basket apparatus, paddle apparatus, or reciprocating cylinder	Dissolution Apparatus 1, Apparatus Type 2, Dissolution Apparatus Type 3
Powders and granules	Flow-through cell (powder/granule sample cell)	Dissolution Apparatus 4
Thin dissolvable films	Basket apparatus and disintegration method	Dissolution Apparatus 1
Chewing gum	Special apparatus (Ph. Eur.)	
Dermal delivery systems (patches)	Paddle over disk, cylinder, and reciprocating holder	Apparatus 5, Apparatus 6, Apparatus 7
Topical (semisolid dosage forms)	Franz cell diffusion system	
Suppositories	Paddle apparatus, modified basket apparatus or dual chamber flow-through cell	Apparatus Type 2, Dissolution Apparatus 1 (modified), Dissolution Apparatus 4 (dual chamber)
Micro-particulate formulations	Modified flow-through cell	Dissolution Apparatus 4 (modified)
Implants	Modified flow-through cell	Dissolution Apparatus 4 (modified)
Aerosols	Cascade impactor	

**Table 2:** Comparison of key features between Franz cell and customised Franz-type cell setup.

Parameters	Franz Cell	Customised Franz-Type Cell Setup
Release surface area	0.78 cm^2^	3.80 cm^2^
Simulation of in vivo motility	Magnetic stirrer	Shaking water bath
Temperature	Can be set (e.g., at 37 °C)	Can be set (e.g., at 37 °C)
Number of samples per run	5	Up to 20
Likelihood of bubble formation upon sample application	High	Very low
Volume of receptor compartment	Fixed	Flexible, can be small
Capacity to measure release of actives present at low concentration in formulation	Relatively low	Relatively high, depending on size of receptor compartment
Option of customization to specific requirements	Not possible	Possible

**Table 3:** Honey samples including botanical origin.

Botanical Origin	Supplier, Year
WA Manuka Honey 1 (*Leptospermum scoparium*)	Hive and Wellness, 2019
WA Manuka Honey 2 (*Leptospermum scoparium*)	Manuka Life, 2019
WA Coastal Peppermint (*Agonis flexuosa*)	Margaret River Honey Company, 2019
WA Jarrah Honey (*Eucalyptus marginata*)	Hive and Wellness, 2019
New Zealand Manuka Honey (*Leptospermum scoparium)*	Hive and Wellness, 2018

**Table 4:** Release data for selected Jarrah honey constituents (*n* = 3, data represents mean ± SD).

Sample	Components (RF)	% Components of Baseline Released at Different Time Points (h)											
		0.25		0.50		1		3		6		12	
		Franz	New	Franz	New	Franz	New	Franz	New	Franz	New	Franz	New
Jarrah pure honey	0.21	0.00	6.6 ± 1.2	6.3 ± 1.1	13.5 ± 1.2	15.2 ± 1.2	23.4 ± 1.1	48.4 ± 1.3	60.3 ± 0.9	79.2 ± 1.4	86.2 ± 1.3	99.2 ± 0.8	99.3 ± 0.6
	0.53	0.00	7.3 ± 1.2	6.4 ± 1.1	14.6 ± 1.1	16.2 ± 1.4	24.7 ± 1.3	49.5 ± 1.0	59.2 ± 1.0	81.2 ± 1.2	88.2 ± 1.2	99.3 ± 0.8	99.2 ± 0.7
Jarrah pre-gel	0.21	0.00	6.3 ± 1.0	6.5 ± 1.2	13.4 ± 1.2	15.4 ± 1.1	23.6 ± 1.4	48.4 ± 1.4	58.2 ± 0.9	79.1 ± 1.0	86.3 ± 1.2	99.3 ± 0.7	99.3 ± 0.6
	0.53	0.00	7.5 ± 1.2	6.5 ± 1.1	14.7 ± 1.1	16.2 ± 1.4	24.2 ± 1.3	48.2 ± 1.2	58.7 ± 1.2	81.3 ± 0.9	88.3 ± 1.0	99.3 ± 0.8	99.3 ± 0.7
WA Manuka 1 pure honey	0.38	0.00	6.4 ± 1.1	6.5 ± 1.1	13.4 ± 1.1	15.5 ± 1.1	23.7 ± 1.2	48.4 ± 1.2	58.3 ± 0.9	79.2 ± 1.1	86.3 ± 1.1	99.4 ± 0.8	99.5 ± 0.7
WA Manuka 1 pre-gel	0.38	0.00	6.3 ± 10.	6.6 ± 1.1	13.5 ± 1.3	15.4 ± 1.1	23.7 ± 1.2	48.4 ± 1.2	58.8 ± 1.0	79.2 ± 1.0	86.5 ± 1.2	99.4 ± 0.7	99.4 ± 0.7
WA Manuka 2 pure honey	0.38	0.00	7.6 ± 1.1	6.6 ± 1.1	14.8 ± 1.1	16.3 ± 1.2	24.3 ± 1.3	48.3 ± 1.2	58.7 ± 1.1	81.4 ± 1.0	88.4 ± 1.1	99.4 ± 1.1	99.4 ± 0.9
WA Manuka 2 pre-gel	0.38	0.00	7.5 ± 1.0	6.4 ± 1.1	14.6 ± 1.2	16.4 ± 1.1	24.4 ± 1.1	48.6 ± 1.3	58.7 ± 1.2	81.6 ± 0.9	88.5 ± 1.1	99.4 ± 0.9	99.4 ± 1.0
CP pure honey	0.20	0.00	7.3 ± 1.0	7.5 ± 1.0	14.4 ± 1.1	15.5 ± 1.1	24.6 ± 1.0	49.4 ± 1.2	59.1 ± 0.9	79.8 ± 1.0	87.3 ± 1.0	99.2 ± 0.9	99.2 ± 0.8
	0.53	0.00	7.8 ± 1.1	7.6 ± 1.1	15.2 ± 1.1	17.3 ± 1.2	25.2 ± 1.3	48.9 ± 1.2	59.0 ± 1.1	82.1 ± 1.0	89.2 ± 1.1	99.3 ± 0.7	99.4 ± 0.7
CP pre-gel	0.20	0.00	6.3 ± 1.0	6.6 ± 1.1	13.5 ± 1.3	15.4 ± 1.1	23.7 ± 1.2	48.4 ± 1.2	58.8 ± 1.0	79.2 ± 1.0	86.5 ± 1.2	99.4 ± 0.7	99.4 ± 0.8
	0.53	0.00	7.8 ± 1.1	7.3 ± 1.2	16.1 ± 1.2	17.2 ± 1.1	25.3 ± 1.1	49.1 ± 1.2	59.2 ± 1.1	82.4 ± 0.9	89.4 ± 1.1	99.5 ± 0.9	99.5 ± 0.7
NZ Manuka pure honey	0.32	0.00	7.4 ± 1.0	7.5 ± 1.0	14.4 ± 1.1	15.4 ± 1.1	24.6 ± 1.0	49.3 ± 1.2	59.1 ± 0.9	79.8 ± 1.0	87.4 ± 1.0	99.3 ± 0.9	99.3 ± 0.8
	0.39	0.00	7.7 ± 1.1	7.5 ± 1.1	15.4 ± 1.1	17.3 ± 1.2	25.3 ± 1.3	49.0 ± 1.2	58.9 ± 1.1	82.5 ± 1.0	89.4 ± 1.1	99.4 ± 0.7	99.4 ± 0.7
NZ Manuka pre-gel	0.32	0.00	6.3 ± 1.1	6.3 ± 1.1	13.4 ± 1.0	15.7 ± 1.1	23.8 ± 1.0	48.5 ± 1.2	58.7 ± 1.0	79.3 ± 1.1	86.6 ± 1.2	99.6 ± 0.7	99.5 ± 0.8
	0.39	0.00	7.8 ± 1.1	7.3 ± 1.0	16.2 ± 1.2	17.2 ± 1.0	25.3 ± 1.1	49.2 ± 1.2	59.2 ± 1.0	82.4 ± 0.9	89.4 ± 1.0	99.6 ± 0.8	99.7 ± 0.8

**Table 5:** % components released per unit area of the dialysis membrane (*n* = 3, data represents mean ± SD).

Sample	Components (RF)	% Released per Unit Area of the Dialysis Membrane											
		0.25		0.50		1		3		6		12	
		Franz	New	Franz	New	Franz	New	Franz	New	Franz	New	Franz	New
Jarrah pure honey	0.21	0	1.7 ±0.5	8.1 ± 0.5	3.6 ± 0.6	19.5 ± 0.4	6.2 ± 0.5	62.0 ± 0.7	15.9 ± 0.6	101.6 ± 0.6	22.7 ± 0.6	127.2 ± 0.4	26.1 ± 0.6
	0.53	0	1.9 ± 0.5	8.2 ± 0.5	3.8 ± 0.5	20.8 ± 0.5	6.5 ± 0.5	63.5 ± 0.4	15.6 ± 0.4	104.1 ± 0.5	23.2 ± 0.5	127.3 ± 0.5	26.1 ± 0.5
Jarrah pre-gel	0.21	0	1.7 ± 0.5	8.3 ± 0.4	3.5 ± 0.5	19.8 ± 0.6	6.2 ± 0.6	62.0 ± 0.5	15.3 ± 0.5	101.5 ± 0.5	22.7 ± 0.4	127.3 ± 0.6	26.1 ± 0.5
	0.53	0	2.0 ± 0.5	8.4 ± 0.5	3.9 ± 0.5	20.8 ± 0.5	6.4 ± 0.5	61.8 ± 0.5	15.5 ± 0.6	104.2 ± 0.6	23.2 ± 0.6	127.3 ± 0.5	26.1 ± 0.4
WA Manuka 1 pure honey	0.38	0	1.7 ± 0.4	8.4 ± 0.6	3.5 ± 0.4	19.8 ± 0.4	6.2 ± 0.4	62.0 ± 0.6	15.3 ± 0.7	101.5 ± 0.4	22.7 ± 0.5	127.5 ± 0.4	26.2 ± 0.6
WA Manuka 1 pre-gel	0.38	0	1.7 ± 0.5	8.5 ± 0.5	3.6 ± 0.6	17.2 ± 0.6	6.2 ± 0.5	62.1 ± 0.5	15.5 ± 0.7	101.6 ± 0.6	22.8 ± 0.5	127.4 ± 0.5	26.1 ± 0.6
WA Manuka 2 pure honey	0.38	0	2.0 ± 0.5	8.4 ± 0.4	3.9 ± 0.5	20.9 ± 0.5	6.4 ± 0.5	62.0 ± 0.6	15.5 ± 0.4	104.4 ± 0.5	23.3 ± 0.5	127.5 ± 0.5	26.2 ± 0.6
WA Manuka 2 pre-gel	0.38	0	2.0 ± 0.6	8.2 ± 0.6	3.9 ± 0.5	21.0 ± 0.4	6.4 ± 0.6	62.3 ± 0.5	15.4 ± 0.6	104.6 ± 0.5	23.3 ± 0.5	127.4 ± 0.6	26.1 ± 0.4
CP pure honey	0.20	0	1.9 ± 0.4	9.7 ± 0.5	3.8 ± 0.5	19.8 ± 0.4	6.5 ± 0.4	63.3 ± 0.5	15.6 ± 0.6	102.3 ± 0.5	23.0 ± 0.6	127.2 ± 0.5	26.1 ± 0.5
	0.53	0	2.0 ± 0.5	9.7 ± 0.5	4.0 ± 0.6	22.2 ± 0.6	6.6 ± 0.4	62.7 ± 0.4	15.5 ± 0.5	105.3 ± 0.5	23.5 ± 0.4	127.4 ± 0.4	26.2 ± 0.6
CP pre-gel	0.20	0	1.7 ± 0.5	8.5 ± 0.4	3.6 ± 0.6	19.7 ± 0.5	6.2 ± ±0.7	62.1 ± 0.5	15.5 ± 0.4	101.6 ± 0.6	22.8 ± 0.6	127.4 ± 0.5	26.1 ± 0.5
	0.53	0	2.1 ± 0.5	9.4 ± 0.5	4.2 ± 0.5	22.1 ± 0.5	6.7 ± 0.4	63.0 ± 0.4	15.6 ± 0.5	105.7 ± 0.4	23.5 ± 0.6	127.6 ± 0.5	26.2 ± 0.5
NZ Manuka pure honey	0.32	0	1.9 ± 0.5	9.6 ± 0.4	3.8 ± 0.4	19.8 ± 0.5	6.5 ± 0.6	63.2 ± 0.5	15.6 ± 0.6	102.3 ± 0.5	23.0 ± 0.4	127.3 ± 0.4	26.1 ± 0.4
	0.39	0	2.0 ± 0.6	9.6 ± 0.5	4.1 ± 0.5	22.2 ± 0.4	6.6 ± 0.7	62.8 ± 0.4	15.5 ± 0.5	105.8 ± 0.6	23.5 ± 0.4	127.5 ± 0.5	26.2 ± 0.6
NZ Manuka pre-gel	0.32	0	1.7 ± 0.4	8.1 ± 0.5	3.5 ± 0.6	20.1 ± 0.4	6.3 ± 0.7	62.1 ± 0.5	15.5 ± 0.4	101.6 ± 0.6	22.8 ± 0.5	127.6 ± 0.5	26.2 ± 0.5
	0.39	0	2.1 ± 0.5	9.4 ± 0.4	4.3 ± 0.4	22.1 ± 0.5	6.7 ± 0.3	63.0 ± 0.4	15.6 ± 0.6	105.7 ± 0.4	23.5 ± 0.5	127.8 ± 0.6	26.2 ± 0.5

**Table 6:** Release rate of honey constituents (*n* = 3, data represents mean ± SD).

Sample	Component of Interest (Presented by R_F_ Values)	Time (h) Required to Release 25, 50, and 75%					
		T25%		T50%		T75%	
		Franz	New	Franz	New	Franz	New
Pure JAR honey extract	0.21	1.6 ± 1.1	1.1 ± 1.2	3.1 ± 1.3	2.5 ± 1.2	5.7 ± 1.0	5.2 ± 1.2
	0.53	1.5 ± 1.2	1.0 ± 1.1	3.0 ± 1.1	2.5 ± 1.1	5.5 ± 1.1	5.1 ± 1.1
JAR pre-gel extract	0.21	1.6 ± 1.0	1.1 ± 1.2	3.1 ± 1.0	2.6 ± 1.1	5.7 ± 1.2	5.2 ± 1.3
	0.53	1.5 ± 1.2	1.0 ± 1.1	3.1 ± 1.2	2.5 ± 1.2	5.5 ± 1.1	5.1 ± 1.0
WA Manuka 1 pure honey	0.38	1.6 ± 1.2	1.1 ± 1.1	3.1 ± 1.2	2.6 ± 1.1	5.7 ± 1.1	5.2 ± 1.0
WA Manuka 1 pre-gel	0.38	1.6 ± 1.1	1.0 ± 1.1	3.1 ± 1.1	2.5 ± 1.1	5.7 ± 1.1	5.2 ± 1.1
WA Manuka 2 pure honey	0.38	1.5 ± 1.2	1.0 ± 1.2	3.1 ± 1.1	2.5 ± 1.1	5.5 ± 1.1	5.1 ± 1.1
WA Manuka 2 pre-gel	0.38	1.5 ± 1.2	1.0 ± 1.2	3.1 ± 1.2	2.6 ± 1.2	5.5 ± 1.1	5.1 ± 1.1
CP pure honey	0.20	1.6 ± 1.1	1.0 ± 1.2	3.0 ± 1.1	2.5 ± 1.2	5.6 ± 1.0	5.1 ± 1.2
	0.53	1.4 ± 1.2	1.0 ± 1.1	3.1 ± 1.1	2.5 ± 1.1	5.5 ± 1.1	5.0 ± 1.1
CP pre-gel	0.20	1.6 ± 1.1	1.0 ± 1.2	3.1 ± 1.1	2.5 ± 1.1	5.7 ± 1.2	5.2 ± 1.1
	0.53	1.4 ± 1.2	1.0 ± 1.1	3.0 ± 1.2	2.5 ± 1.2	5.5 ± 1.1	5.0 ± 1.1
NZ Manuka pure honey	0.32	1.6 ± 1.1	1.0 ± 1.2	3.0 ± 1.2	2.5 ± 1.2	5.6 ± 1.1	5.1 ± 1.2
	0.39	1.4 ± 1.2	1.0 ± 1.1	3.1 ± 1.1	2.5 ± 1.1	5.4 ± 1.1	5.0 ± 1.1
NZ Manuka pre-gel	0.32	1.6 ± 1.1	1.0 ± 1.2	3.1 ± 1.2	2.5 ± 1.1	5.7 ± 1.2	5.2 ± 1.2
	0.39	1.4 ± 1.2	1.0 ± 1.0	3.0 ± 1.2	2.5 ± 1.2	5.5 ± 1.1	5.0 ± 1.1

## Data Availability

Not applicable.

## Supplementary Materials

The following supporting information can be downloaded at https://www.mdpi.com/article/10.3390/mps6040070/s1. Figure S1: Peak profile of compounds of interest (R_F_ 0.38) released from pure WA Manuka honey 1 using Franz cell and customised Franz-type cell setup: (a) 15 min; (b) 30 min; (c) 1 h; (d) 3 h; (e) 6 h; (f) 12 h; (g) baseline (0 min). Figure S2: Peak profile of compounds of interest (R_F_ 0.38) released from WA Manuka honey 1 pre-gel solution using Franz cell and customised Franz-type cell setup: (a) 15 min; (b) 30 min; (c) 1 h; (d) 3 h; (e) 6 h; (f) 12 h; (g) baseline (0 min). Figure S3: Peak profile of compounds of interest (R_F_ 0.38) released from pure WA Manuka honey 2 honey using Franz cell and customised Franz-type cell setup: (a) 15 min; (b) 30 min; (c) 1 h; (d) 3 h; (e) 6 h; (f) 12 h; (g) baseline (0 min). Figure S4: Peak profile of compounds of interest (R_F_ 0.38) released from WA Manuka honey 2 pre-gel solution using Franz cell and customised Franz-type cell setup: (a) 15 min; (b) 30 min; (c) 1 h; (d) 3 h; (e) 6 h; (f) 12 h; (g) baseline (0 min). Figure S5: Peak profile of compounds of interest (R_F_ 0.20 and R_F_ 0.53) released from pure Coastal Peppermint honey using Franz cell and customised Franz-type cell setup: (a) 15 min; (b) 30 min; (c) 1 h; (d) 3 h; (e) 6 h; (f) 12 h; (g) baseline (0 min). Figure S6: Peak profile of compounds of interest (R_F_ 0.20 and R_F_ 0.53) released from Coastal Peppermint honey pre-gel solution using Franz cell and customised Franz-type cell setup: (a) 15 min; (b) 30 min; (c) 1 h; (d) 3 h; (e) 6 h; (f) 12 h; and (g) baseline (0 min). Figure S7: Peak profile of compounds of interest (R_F_ 0.32 and R_F_ 0.39) released from pure NZ Manuka honey using Franz cell and customised Franz-type cell setup at (a) 15 min; (b) 30 min; (c) 1 h; (d) 3 h; (e) 6 h; (f); 12 h; (g) baseline (0 min). Figure S8: Peak profile of compounds of interest (R_F_ 0.32 and R_F_ 0.39) released from NZ Manuka honey pre-gel solution using Franz cell and customised Franz-type cell setup: (a) 15 min; (b) 30 min; (c) 1 h; (d) 3 h; (e) 6 h; (f) 12 h; (g) baseline (0 min).
