# Supplementary material for: Application of a Customised Franz-Type Cell Coupled with HPTLC to Monitor the Timed Release of Bioactive Components in Complex Honey Matrices
Source: Methods Protoc. 2023 Aug 3;6(4):70. doi: 10.3390/mps6040070 (PMC10459218; doi:10.3390/mps6040070)
Supplement: Supplementary file 1 [file mps-06-00070-s001.zip › mps-2484549-supplementary.pdf]

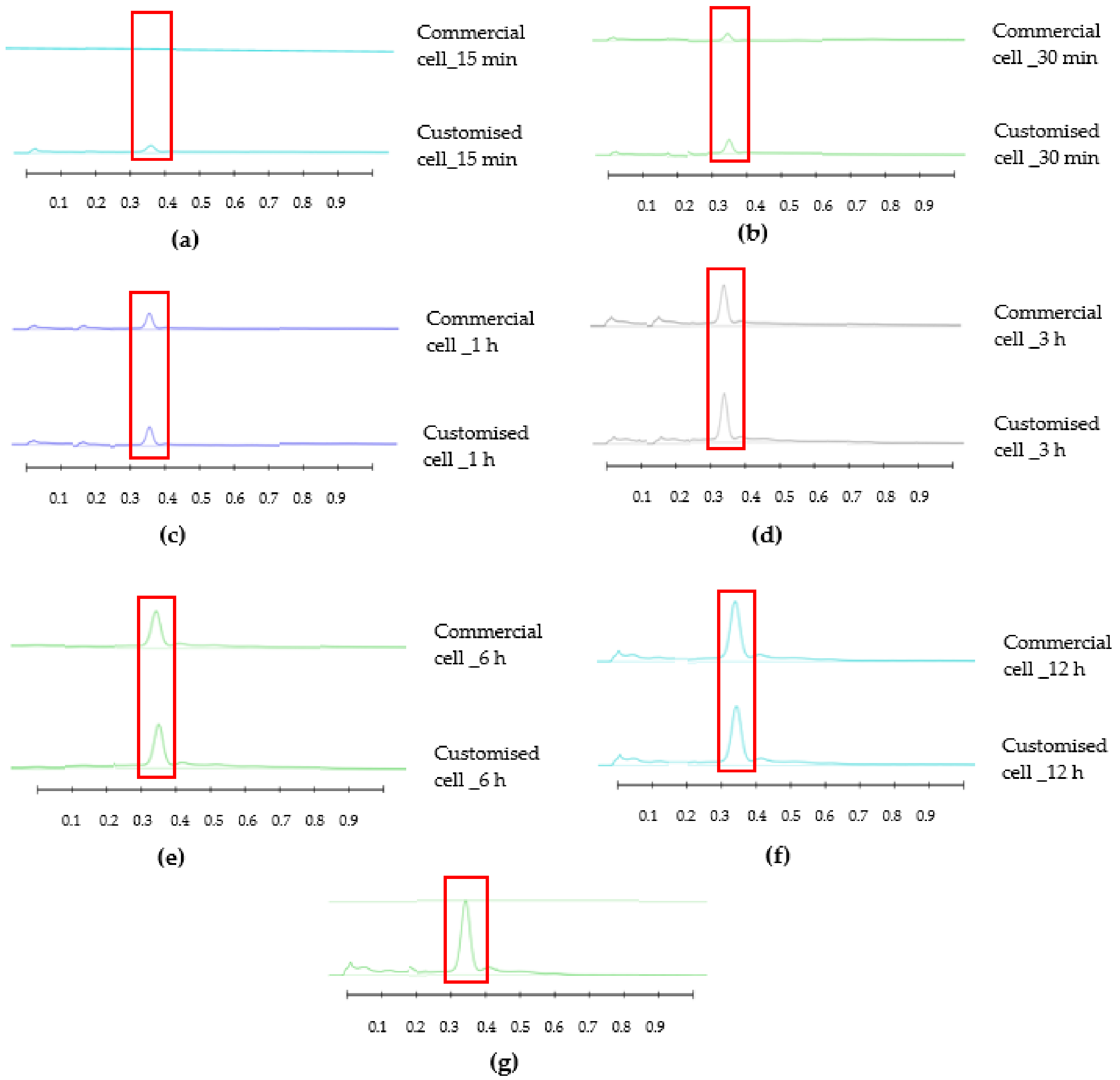


**Figure S1.** Peak profile of compounds of interest ($R_F$ 0.38) released from pure WA Manuka honey 1 honey using Franz cell and customised Franz-type cell set-up at (a) 15 min, (b) 30 min, (c) 1 h, (d) 3 h, (e) 6 h, (f) 12 h and (g) baseline (0 min). Red box highlights monitored band.

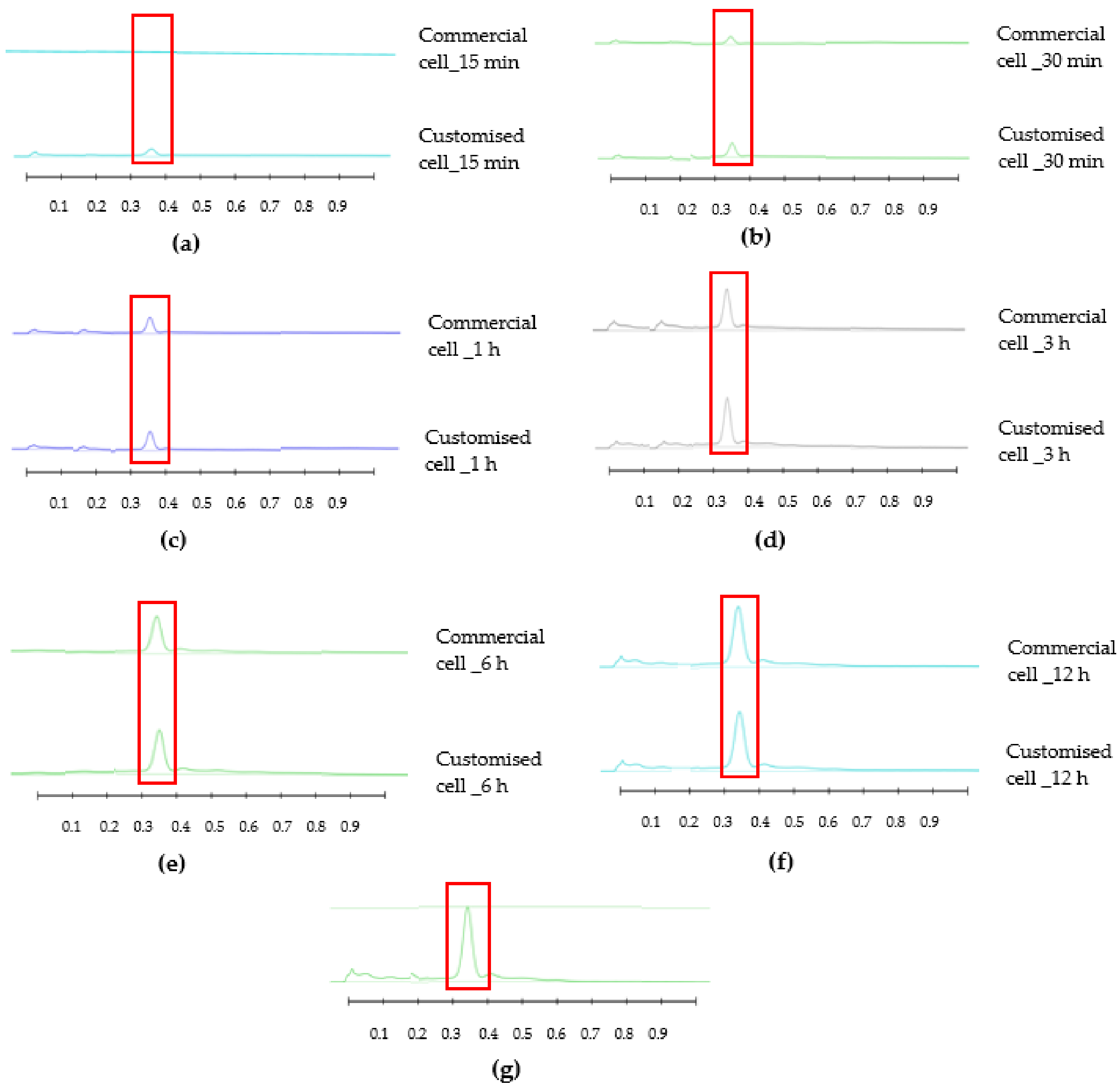


**Figure S2.** Peak profile of compounds of interest ($R_F$ 0.38) released from WA Manuka honey 1 pre-gel solution using Franz cell and customised Franz-type cell set-up at (a) 15 min, (b) 30 min, (c) 1 h, (d) 3 h, (e) 6 h, (f) 12 h and (g) baseline (0 min). Red box highlights monitored band.

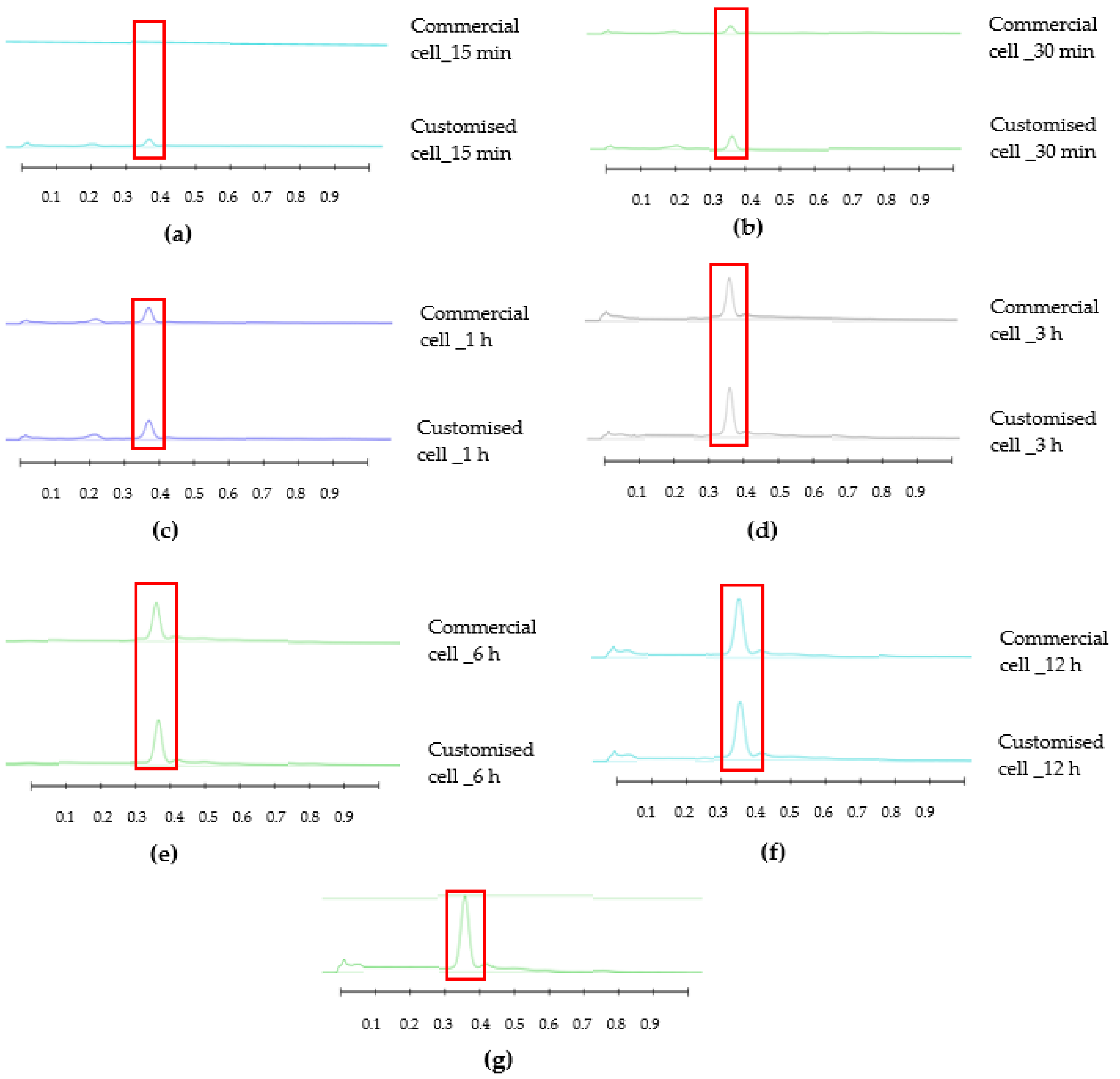


**Figure S3.** Peak profile of compounds of interest ($R_F$ 0.38) released from pure WA Manuka honey 2 honey using Franz cell and customised Franz-type cell set-up at (a) 15 min, (b) 30 min, (c) 1 h, (d) 3 h, (e) 6 h, (f) 12 h and (g) baseline (0 min). Red box highlights monitored band.

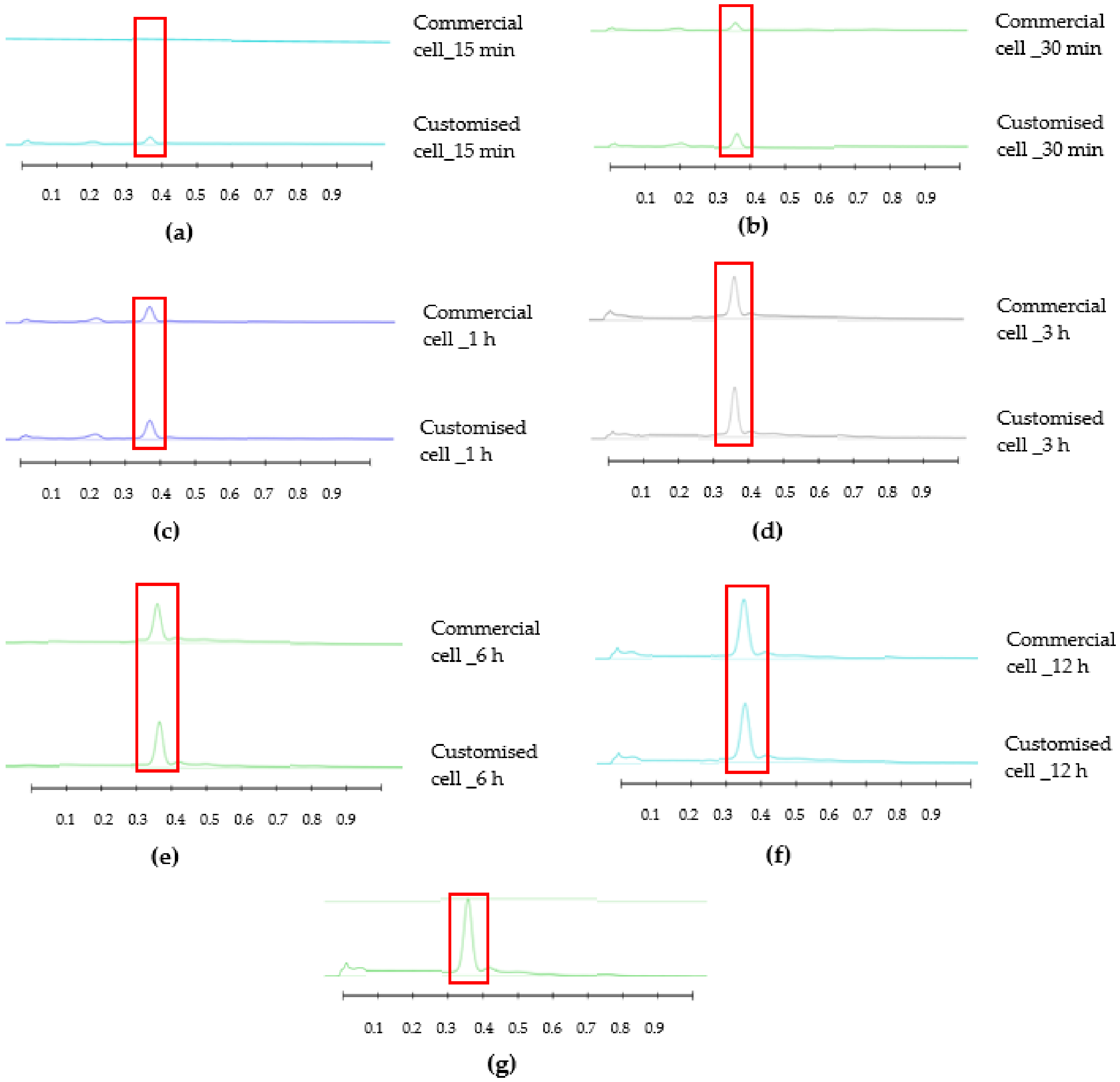


**Figure S4.** Peak profile of compounds of interest ($R_F$ 0.38) released from WA Manuka honey 2 pre-gel solution using Franz cell and customised Franz-type cell set-up at (a) 15 min, (b) 30 min, (c) 1 h, (d) 3 h, (e) 6 h, (f) 12 h and (g) baseline (0 min). Red box highlights monitored band.

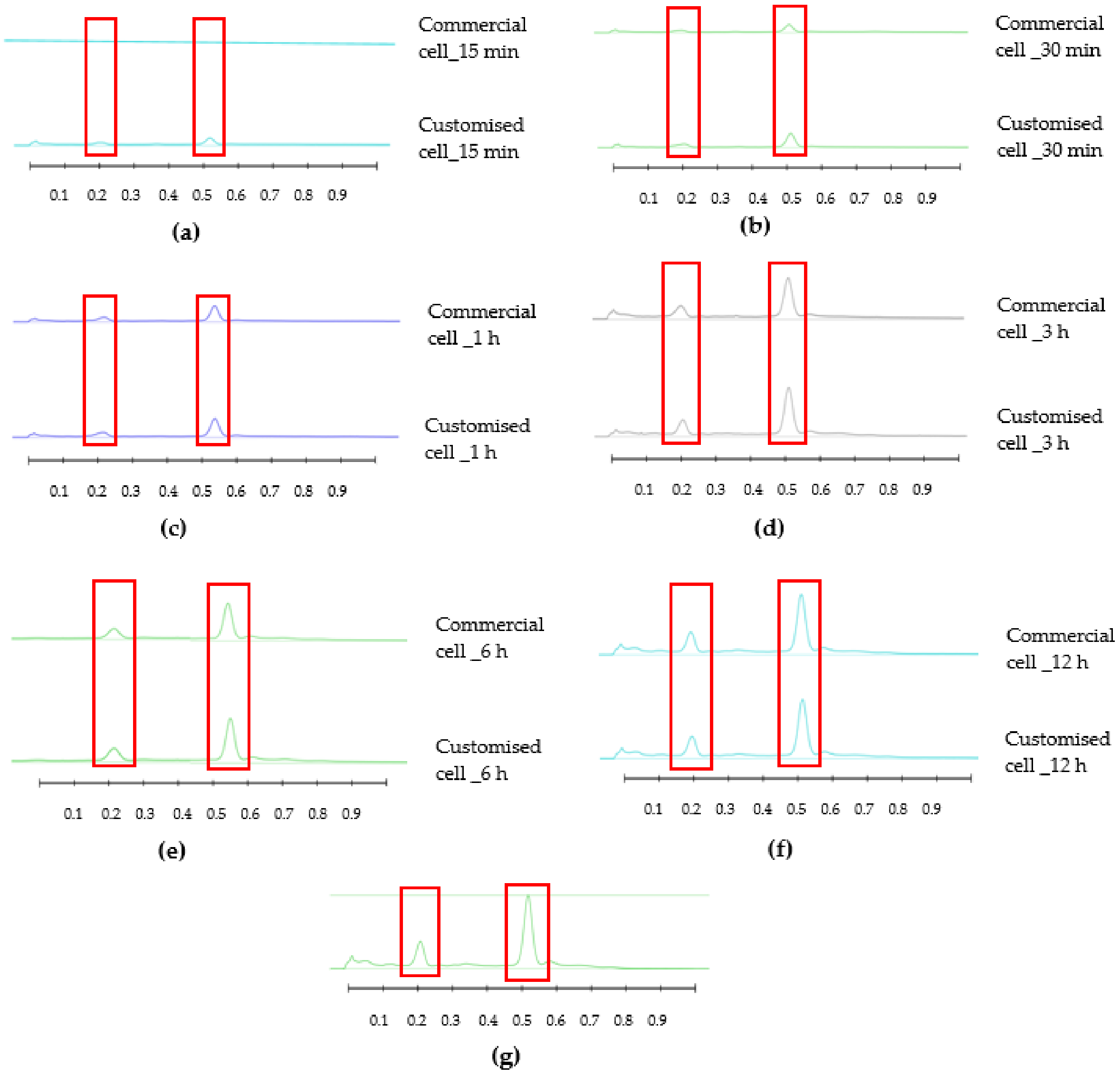


**Figure S5.** Peak profile of compounds of interest ($R_F$ 0.20 and $R_F$ 0.53) released from pure Coastal Peppermint honey using Franz cell and customised Franz-type cell set-up at (a) 15 min, (b) 30 min, (c) 1 h, (d) 3 h, (e) 6 h, (f) 12 h and (g) baseline (0 min). Red boxes highlight monitored bands.

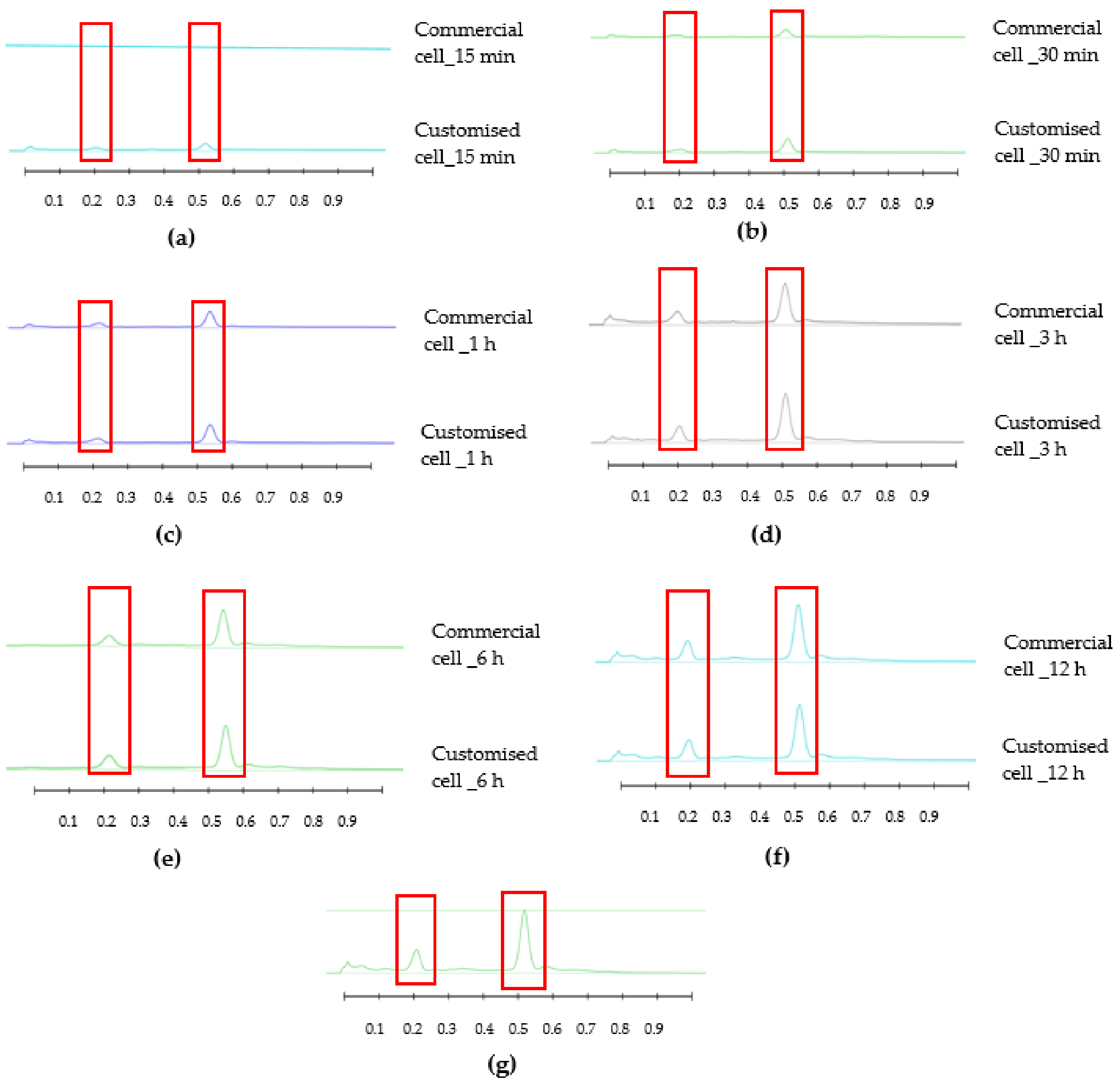


**Figure S6.** Peak profile of compounds of interest ($R_F$ 0.20 and $R_F$ 0.53) released from Coastal Peppermint honey pre-gel solution using Franz cell and customised Franz-type cell set-up at (a) 15 min, (b) 30 min, (c) 1 h, (d) 3 h, (e) 6 h, (f) 12 h and (g) baseline (0 min). Red boxes highlight monitored bands.

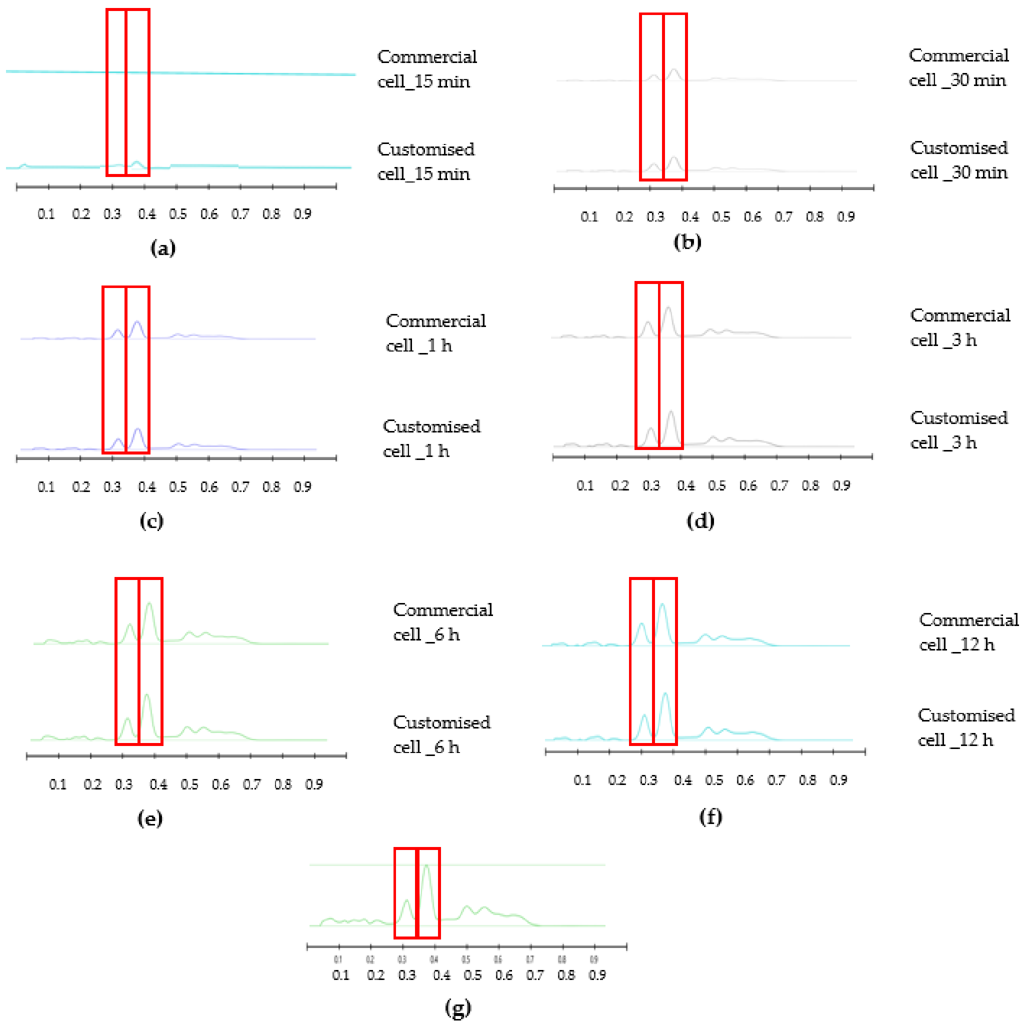


**Figure S7.** Peak profile of compounds of interest ($R_F$ 0.32 and $R_F$ 0.39) released from pure NZ Manuka honey using Franz cell and customised Franz-type cell set-up at (a) 15 min, (b) 30 min, (c) 1 h, (d) 3 h, (e) 6 h, (f) 12 h and (g) baseline (0 min). Red boxes highlight monitored bands.

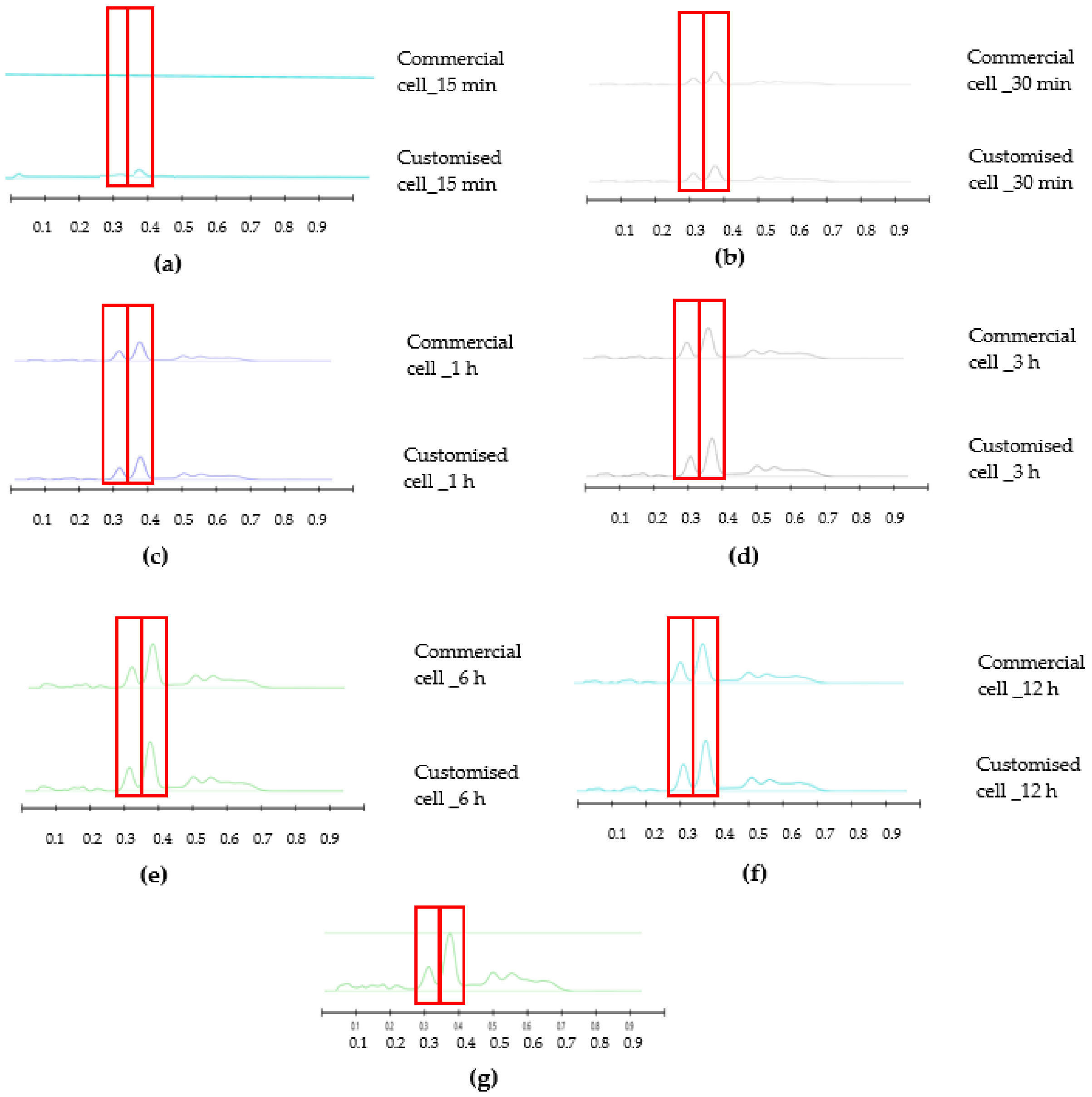


**Figure S8.** Peak profile of compounds of interest ($R_F$ 0.32 and $R_F$ 0.39) released from NZ Manuka honey pre-gel solution using Franz cell and customised Franz-type cell set-up at (a) 15 min, (b) 30 min, (c) 1 h, (d) 3 h, (e) 6 h, (f) 12 h and (g) baseline (0 min). Red boxes highlight monitored bands.
